# Antarctic root endophytes improve physiological performance and yield in crops under salt stress by enhanced energy production and Na^+^ sequestration

**DOI:** 10.1038/s41598-020-62544-4

**Published:** 2020-04-02

**Authors:** Marco A. Molina-Montenegro, Ian S. Acuña-Rodríguez, Cristian Torres-Díaz, Pedro E. Gundel, Ingo Dreyer

**Affiliations:** 1 Instituto de Ciencias Biológicas, Universidad de Talca, Campus Talca, Chile; 2 Centro de Estudios Avanzados en Zonas Áridas (CEAZA), Universidad Católica del Norte, Coquimbo, Chile; 3 Centro de Investigaciones y Estudios Avanzados del Maule (CIEAM), Universidad Católica del Maule, Talca, Chile; 4 Grupo de Biodiversidad y Cambio Global (BCG), Departamento de Ciencias Básicas, Universidad del Bío-Bío, Chillán, Chile; 5 IFEVA, Universidad de Buenos Aires, CONICET, Facultad de Agronomía, Buenos Aires, Argentina; 6 Centro de Bioinformática y Simulación Molecular (CBSM), Facultad de Ingeniería, Universidad de Talca, Campus Talca, Chile

**Keywords:** Microbiology, Physiology, Plant sciences

## Abstract

Climatic change is pointed as one of the major challenges for global food security. Based on current models of climate change, reduction in precipitations and in turn, increase in the soil salinity will be a sharp constraint for crops productivity worldwide. In this context, root fungi appear as a new strategy to improve plant ecophysiological performance and crop yield under abiotic stress. In this study, we evaluated the impact of the two fungal endophytes *Penicillium brevicompactum* and *P. chrysogenum* isolated from Antarctic plants on nutrients and Na^+^ contents, net photosynthesis, water use efficiency, yield and survival in tomato and lettuce, facing salinity stress conditions. Inoculation of plant roots with fungal endophytes resulted in greater fresh and dry biomass production, and an enhanced survival rate under salt conditions. Inoculation of plants with the fungal endophytes was related with a higher up/down-regulation of ion homeostasis by enhanced expression of the *NHX1* gene. The two endophytes diminished the effects of salt stress in tomato and lettuce, provoked a higher efficiency in photosynthetic energy production and an improved sequestration of Na^+^ in vacuoles is suggested by the upregulating of the expression of vacuolar NHX1 Na^+^/H^+^ antiporters. Promoting plant-beneficial interactions with root symbionts appears to be an environmentally friendly strategy to mitigate the impact of climate change variables on crop production.

## Introduction

The earth faces dramatic environmental changes caused by the living habits of an increasing human population^1–3^ which has consequences on life quality. A combination of effects from man-made global climate change, land degradation and contamination may compromise food production, for instance. Although traditional breeding and biotechnology are likely to overcome part of these constraints by engineering plants matching the environment^4^, more ecological and friendly practices would be highly beneficial as means to reduce the use of agro-pesticides and/or to enhance the environmental tolerance in agroecosystems. In this context, microbial symbionts of plants appear a promising alternative for improving plant performance and maintaining, or even increasing, the yield of crops^5–11^.

Soil salinization as a result of inappropriate cultural practices and excessive agricultural use is a symptom of land degradation. It currently affects a vast territory of productive areas throughout the world^12–14^. Additionally, agriculture expands to regions that are naturally affected by salinity. Therefore, plant resistance to salt, mainly to the sodium cation (Na^+^), is a desirable trait in cultivated plants. One mechanism of plant tolerance in glycophytic (i.e. salt susceptible) species consists of reducing the cytoplasmic sodium concentration by sequestering Na^+^ in the vacuole via tonoplast Na^+^/H^+^ antiporters^15,16^. Apart from reducing sodium toxicity, increased ion concentrations in vacuoles allow plants to alleviate water deficit and maintain positive carbon gain^15,17^. The Na^+^ transport is governed by a family of genes of the *NHX*-type, well described in *Arabidopsis thaliana* but also present in other crop species^7,10,11,18^. Tomato plants are able to accumulate Na^+^ in leaves when growing in soils with high sodium contents^19^ and an increased tolerance has been observed in plants overexpressing *NHX* genes^20,21^. It has also been scrutinized how plant tolerance to sodium can be improved by the association with root microorganisms^22,23^. For example, inoculation with arbuscular mycorrhizal fungi (AMF) significantly improved yield in tomato under high salt conditions^24,25^. In this case, the fungus supports the exclusion of Na^+^ from the plants. In both, saline and no-saline soils, AMF-colonized plants had a lower Na^+^ concentration than in non-colonized plants^26^. Root symbiotic microorganisms seem to activate the immune system of plants and increase overall performance, even in saline soils. It is unknown, however, in which way the presence of (a) root symbiont(s) cause(s) reduced Na^+^ toxicity.

The association of plants with multiple microorganisms (e.g., fungal endophytes) either in roots or aboveground tissues is not the exception but the rule^26–29^. Some of the often cited effects of fungal endophytes are the gain of new metabolic capabilities by the host, the provision with secondary metabolites, and the stimulation of plant defense systems against abiotic stress factors and enemies^8,30–33^. For example, tomato plants (*Solanum lycopersicum*) that were grown on native soils showed an elicited immune response related to protection against oxidative stress compared to conspecific plants grown on sterile soils^34^. This effect was partially reverted when the plants growing in sterile soils were inoculated with the AMF *Funneliformis mosseae*^34^. Arbuscular mycorrhizal fungi also improved the general performance of *Robinia pseudoacacia* seedlings under salt stress^35^. The overall physiological status of the plants (photosynthetic rate, photosystem II quantum efficiency and relative water content) was boosted by the symbiont, and genes encoding membrane transport proteins involved in K^+^/Na^+^ homeostasis in roots were upregulated^35^. Although AM fungi are by far the most studied soil microorganisms^33–36^, other root colonizers (e.g., *Trichoderma* spp., *Piriformospora indica*, *Penicillium* spp.) are known to deliver benefits to their host plants, too. Strains of *Trichoderma*, a common soil fungus, increased the tolerance to Na^+^ in *Arabidopsis thaliana* by increasing the level of the auxin IAA (indole-3-acetic acid), the production of osmolites and antioxidants^37^.

Symbiotic fungi are proposed to be beneficial for the adaptation of plants to environmental stress factors^33,38–40^. Different bio-prospective research programs started to consider fungi present in harsh environments as an unexplored source to find new metabolic pathways and ultimately, new bioactive constituents^41^. In this context, Antarctica has truly unique ecosystems representing one of the most severe climatic conditions for life on earth; i.e., low water availability, high UV-B radiation, extreme low temperatures and saline soils^42^. Nevertheless, there are plants and microorganisms that inhabit this harsh environment. Interestingly, in some cases these microorganisms have been found already to improve plant adaptation to stressful conditions by establishing a so-called ‘functional symbiosis’^43^. Indeed, using the Antarctic plant *Colobanthus quitensis* as a source, we recently identified two species of *Penicillium* that retained their functional role observed in their native plant host even when exposed to a new plant species^7,11^. Thus, considering the geographic isolation and inhospitable conditions for growth that prevails in Antarctica and the referred ecological role of some Antarctic plant endophytes, it is highly plausible that these microorganisms may have unique metabolic pathways with an unexplored potential as biotechnological tools. Antarctic endophytes are thus an interesting new source for strategies using microbial plant symbionts for the adaptation of crops to the large environmental alterations predicted for the ongoing climate change^44^.

In this article, we report the beneficial effect of fungal endophytes isolated from roots of plants native to the Antarctic continent on the individual performance and final yield of two crop species, *Lactuca sativa* and *Solanum lycopersicum*, under salt stress. In order to get an insight into the underlying mechanisms, we assessed whether the endophyte-mediated improvement of host plant performance under sodium stress is related with enhanced individual physiological processes such as photosynthesis and water use efficiency, as well as with an upregulation of vacuolar NHX H^+^/Na^+^ antiporters. Finally, we demonstrated that the improved energy production goes hand in hand with an improved pathway for vacuolar Na^+^ sequestration, suggesting an astonishing engineering capacity by Antarctic endophytes.

## Methodology

### Inoculation by fungal endophytes

We used a mix of two Antarctic fungal endophytes (AFE) isolated from the roots of two Antarctic plants, *Colobanthus quitensis* (AFE001) and *Deschampsia antarctica* (AFE002), during the growing season 2016–2017. Details concerning the procedures of fungal isolation, molecular characterization and species identification of these two fungal endophytes can be found in a previous study^7^. The isolates AFE001 (Genebank accession number: KJ881370) and AFE002 (Genebank accession number: KJ881371) were identified as *Penicillium brevicompactum* and *P. chrysogenum*, respectively^7^. These inoculums are maintained as part of the collection of microorganisms in the Plant Ecology Laboratory, Universidad de Talca, Chile. The inoculums were separated in different Petri dishes and then frozen until they were used for the experiments. We used these inoculums in combination because they are the most abundant endophytes found in the two Antarctic vascular plant species.

Fresh inoculums were obtained in March 2017 from single-conidia of AFE001 and AFE002 cultured on potato dextrose agar (PDA) medium diluted eight times and supplemented with 50 mg/ml of streptomycin. Cultures with endophytes were incubated at 22 ± 2 °C and 350 μmol m^−2^ s^−1^ with a photoperiod 14/10 day/night. After two weeks of incubation, conidia were harvested from plates by adding 10 ml of sterile water and gently scraping off conidia with a sterile glass slide. The conidia suspension was adjusted to 100 ml of 0.05% Tween-100, sterilized solution, filtered through three layers of sterile cotton cheesecloth gauze. Conidia concentration was estimated by using a Neubauer chamber and adjusted to 1 × 10^5^ conidia/ml and its viability was tested according to the methodology described by Greenfield *et al*.^45^; the mean conidia viability was >95%.

The endophyte inoculum consisted of a concentrated mix of conidia from the two fungi (*P. brevicompactum* and *P. chrysogenum*). Plants were inoculated with a ~2 ml suspension containing a concentrated mix of spores (5000 spores ml^−1^) from each fungal endophyte directly applied to the rhizosphere of each individual plant. This procedure was repeated twice (15 days apart) to ensure the fungi to establish an effective association; verification of the symbiosis was evidenced by microscopy (Motic BA 310, with camera Moticam 2500) using root smears re-cultured on plates. Before the beginning of the experiment, two plants of each species/treatment were sacrificed to check microscopically for the presence or absence of endophytes by using a dissection microscope (Motic BA410, Chinese Group, CO., Ltda.) after cleaning the roots in 10% KOH (w/v) and staining with trypan blue in an acid glycerol solution.

### Host plant species and experimental design

*Lactuca sativa* L. (lettuce var. Romaine) and *Solanum lycopersicum* L. (tomato var. Moneymaker) seedlings were obtained from seeds germinated in the glasshouse located at the Universidad de Talca, Talca, Chile (35°24′S; 71°37′W), under semi-controlled environmental conditions of light and temperature (760 ± 96 μmol m^−2^ s^−1^ with a photoperiod of 14/10 day/night, and 22 ± 5 °C). For treatment setup, lettuce and tomato seedlings previously inoculated in the laboratory were transplanted into the field in Chilean springtime of 2018 when individuals presented at least four expanded leaves and 3-cm roots. One-hundred seedlings of each species were randomly assigned to one of the four treatments: (*i*) plants without the endophytes were irrigated with 40 mL of tap water plus 50 mM NaCl per day (Soil Electrical Conductivity: 6.8 ± 1.7 mS cm^−1^), (*ii*) plants with the endophytes were irrigated with 40 mL of tap water plus 50 mM NaCl per day (SEC: 7.5 ± 1.9 mS cm^−1^), (*iii*) plants without the endophytes were irrigated with 40 mL of tap water plus 150 mM NaCl per day (SEC: 17.6 ± 1.2 mS cm^−1^), and (*iv*) plants with the endophytes were irrigated with 40 mL of tap water plus 150 mM NaCl per day (SEC: 17.2 ± 1.5 mS cm^−1^). The irrigation of the plants remained constant during the experiment.

The amount of tap water that is normally added to lettuce and tomato crops in different commercial stations in the Maule region of Chile for this time of the year, ranges from 30 to 45 mL/day per plant. The seedlings were transplanted to the field and distributed in rows with a distance between plants of 0.2 m and a distance between rows of 0.5 m. Each treatment was assigned to independent rows with four rows per each treatment. The soil of the plot is characterized by high content of clay, good drainage, and low levels of salt (49 ± 5 mM NaCl; unpublished data). Each individual was fertilized with 0.2 g L^−1^ of Phostrogen (Solaris, NPK, 14:10:27) every 30 days. The experiment lasted for 90 (lettuce) and 100 (tomato) days, and the measurements were carried out simultaneously in all treatments for both species. Environmental conditions were recorded at midday (12:00–15:00 h) during the whole experimental period. Air temperature and relative humidity was recorded with a data logger (HOBO-Pro v2 U-23) and sunlight was registered with a portable photosynthetic active radiation sensor (Li-190 quantum sensor). During the experiment, daily mean temperature and relative humidity were 21.6 °C (±3.8) and 65% (±12), respectively; while daily mean radiation was 1,422 μmol m^−2^ s^−1^ (±336).

### Crop yield

For each treatment, individuals of lettuce and tomato arranged were extracted from the soil without damaging the root system at the end of the experimental period. Then, the roots were washed carefully without removing them from the stem and left to dry in the shade for 1 h. Total fresh biomass of both, shoots and roots of each individual was determined with a digital electronic scale (Boeco BBL-52; 0.01 g-precision). Taking into account the nature of the commercialized product for each species, the individual average final crop yield was estimated after over-drying at 62 °C for 96 h the complete shoot tissues of each lettuce (i.e. the leaves), and the fruits in the case of tomato plants.

### Plant survival

A total of 200 plants of lettuce and 200 plants of tomato were used for this experiment, where half of them (*n* = 100) were randomly assigned to one of the four treatments described previously as well as to field spatial arrangement. Survival was recorded weekly for each individual in the field for 12 weeks.

### Ecophysiological traits

We assessed plant ecophysiological performance by determining the net photosynthesis rate (A) and transpiration rate (E). These parameters were measured on visually healthy leafs using an infrared gas analyzer (IRGA, Infra-Red Gas Analyzer, CIRAS-2, PP-Systems Haverhill, USA). Additionally, we calculated the water use efficiency (WUE) as the ratio between photosynthetic rate and transpiration (A/E). This parameter has been shown to be a good indicator of plant water stress under contrasting microhabitat conditions since reductions in water availability are paralleled with increases in WUE^46^. The experimental design consisted of 25 individuals for each treatment from a full factorial design with repeated measurements. The three response variables (A, E and WUE) were recorded for the same individual at days 30, 60 and 90 (lettuce) or 100 (tomato).

### Gene expression

Total RNA was extracted from leaves of 5 individuals per species/treatment at 0, 30 and 90 (lettuce) or 100 (tomato) days old plants according to the protocol described elsewhere^47^. The yield and purity of the extracted RNA was checked using UV absorption spectra, whereas its integrity was visually determined by electrophoresis on agarose gels. DNA was removed from RNA samples by using TURBO DNA-free (Applied Biosystems, California, USA). The first strand cDNA was synthesized according to the method described elsewhere^48^. Quantitative PCR (qPCR) was performed according to the manufacturer’s instructions in a final volume of 20 μl containing 12.5 μl of the Fast SYBR Green PCR master mix (Applied Biosystems), 5 pmol of each primer and the cDNA. The *Elongation Factor 1a* (*EF1a*) was used as reference gene to quantify the changes in the relative gene expression (up- or down-regulation) of *NHX1* genes. The following primers were used to amplify the *EF1a*: 5′-GTACGCATGGGTGCTTGACAAACTC-3′ (forward) and 5′-ATCAGCCTGGGAGGTACCAGTAAT-3′ (reverse). *LsNHX1* amplicons (~200 bp) were obtained using the following primers: 5′- GACAGTCCTGGAAAATCT-3′ (forward) and 5′-TGTGCCCTGACCTCGTAAACTGAT-3′ (reverse). *LeNHX* amplicons (~200 bp) were obtained using the following primers: 5′-GCACTTCTGTTGCTGTGAGTTCCA-3′ (forward) and 5′- GGTTATCAGCCCAAACACC-3′ (reverse).The conditions of PCR amplification were: an initial cycle of 30 min at 45 °C and 2 min at 95 °C, followed by 40 cycles of 30 s at 95 °C, 30 s at 60 °C and 2 min at 72 °C, followed by a final step of 10 min at 72 °C. Cycle threshold (C_T_) values were determined according to the 2^ΔΔCT^ method^49^. From the qRT-PCR efficiencies and the crossing point deviation, we estimated the relative expression and fold changes (FC) of the target gene (salt treatments vs. controls), by comparing the target gene and the reference gene (log_2_ transformed) according to the method described elsewhere^50^.

### Nutrient content

At the end of the experiment, the concentrations of elements (N, P, K and Na) and molecules (NO_3_^−^ and NH_4_^+^) in the plant biomass were determined for seven individuals from each treatment and expressed as percentage on dry weight basis. All analyses were conducted in the Laboratory of Nutrient Analysis at the Universidad de Talca, Chile. Shoot nutrient concentrations were determined after dry-ashing (except for nitrogen). NO_3_^−^ and NH_4_^+^ were determined after KCl extraction; P by Bray-1 method; K, and Na after ammonium acetate extraction. N was determined via combustion analysis (CNS-2000 Macro Analyzer, Leco Inc., MI, USA). P, K and Na were measured by ICP-OES (Perkin Elmer Optima 3000DV, Wellesley, MA, USA).

### Statistical analysis

Six response variables related with plant fitness, physiological performance, gene expression and nutritional status were analyzed to describe the role of the endophytes on the biological performance of lettuce and tomato under saline stress. A standard two-way analysis of variance (ANOVA) was used to evaluate the effect of saline stress and endophyte inoculation on the final yield and final Na^+^ foliar content. To evaluate the effect of saline stress and endophyte inoculation on those variables measured along time (photosynthesis, water use efficiency (WUE) and gene expression), we used two-way repeated measures (rm) ANOVAs. Model fitting was performed with the *aov* function from the base R options using the individual nested in time as the random error structure which is allowed for homoscedastic and orthogonal designs. Shoot nutrient concentrations (N, P and K) under the four experimental treatments, control/E−, control/E+, salt added/E−, and salt added/E+, were compared for lettuce and tomato using a one-way ANOVA. For the one-way and two-way standard ANOVAs, *a posteriori* differences between treatments were evaluated using Honest Significant Differences (HSD) Tukey tests. Normality and homogeneity of variance were assessed with Shapiro-Wilks and Barlett tests, respectively^51^. *A posteriori* comparison for the rmANOVA models between experimental groups was performed by the comparison of their Estimated Marginal Means (EMMs) by factor levels as supported by the function pairs in the *emmeans* R-package^52^. The Kaplan-Meier survival functions were derived from the censored data of each experimental group using the survfit function from the *survival* R-package^53^. To determine the effect of the experimental factors (i.e.: endophyte infection and saline stress) on the survival probabilities, a Cox proportional-hazard model analysis was performed for each species data using the coxph function of the same package. Further pairwise comparisons were performed using the Peto and Peto modification of the Gehan-Wilcoxon test as allowed in the pairwise_survdiff function, implemented in the *survminer* R-package^54^. The assumption of proportionality between experimental factors for proportional hazard models was verified with the cox.zph R-function^53^.

## Results

To evaluate the stress-mitigating potential of a combination of two Antarctic fungal endophytes, *Penicillium brevicompactum* and *P. chrysogenum*, they were tested under salt-stress conditions on two crop species, lettuce (*Lactuca sativa*) and tomato (*Solanum lycopersicum*). As in normal agricultural growth conditions plants were irrigated daily with 40 ml of water containing 50 mM (control) and 150 mM NaCl (stress), respectively. This type of experiment was set up with plants inoculated with the two fungi and in the absence of the endophytes. The differently treated plants were compared with respect to crop yield, the leaf nutrient content, water use efficiency, and the net photosynthetic rate.

### Crop yield

**Figure 1:**
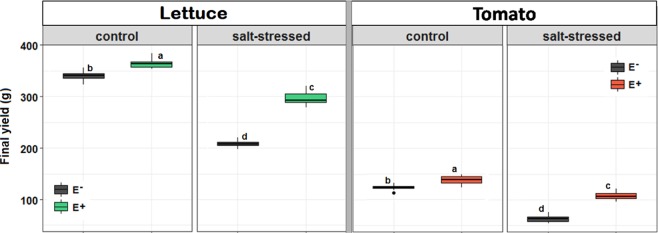
Effect of saline treatment (50 mM NaCl, control, vs. 150 mM NaCl, salt-stressed) and fungal inoculation (E− vs. E+) on the final yield of lettuce (left) and tomato (right) at the end of the experiment. Different lowercase letters indicate, for each species separately, significant differences between treatments (Tukey HSD tests; α = 0.05). Values are means ± SD (*n* = 25).

As expected, salt stress reduced the final yield of both crops drastically (Fig. 1, 50/−E vs. 150/−E, −38% for lettuce; −48% for tomato). This inhibitory effect, however, was strongly mitigated by endophyte inoculation (Fig. 1, 50/−E vs. 150/+E, −13% for both crops). While the presence of the endophytes increased the yield in both control and stress conditions, their impact on final yield was stronger under saline stress. The endophytes provoked a yield increase of +42% in lettuce and of +68% in tomato under salt stress (150/−E vs. 150/+E), while in control conditions (50/−E vs. 50/+E) a rise of +7% (lettuce) and +11% (tomato) was observed, only (Fig. 1).

### Plant survival

**Figure 2:**
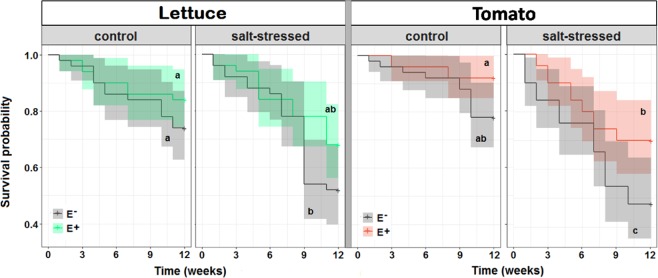
Temporal development of the survival rate of lettuce (left) and tomato plants (right) in the presence (E+) or absence (E−) of endophytes under salt stress (150 mM NaCl) and in control conditions (50 mM NaCl). For each condition, 50 plants were selected and their survival was monitored over time.

Besides the yield increase of the surviving plants also the survival probability improved due to the presence of the endophytes under salt stress. To pinpoint this effect, we described the survival rates in our experiments (Fig. 2) with a Cox proportional hazards model (Table 1). The resulting hazard ratio (HR) described changes in the mortality risk due to the different treatments; HR = 1 means that the treatment did not affect mortality risk, HR > 1 means mortality risk has increased, HR < 1 means that mortality risk has decreased. The Cox regression estimated that the hazard ratios of the applied salt stress were 2.2 ± 0.7 (lettuce) and 3.3 ± 1.1 (tomato), respectively. This means that the salt stress significantly increased the mortality rates of the plants. When the salt stress was applied in combination with endophyte inoculation, however, the hazard ratios were not significantly different from 1 (1.2 ± 0.6 for lettuce and 1.7 ± 1.0 for tomato). Thus, the endophytes compensated for the salt stress resulting in an unchanged mortality rate. In low salt conditions, inoculation with the endophytes resulted in hazard ratios of 0.7 ± 0.3 (lettuce) and 0.4 ± 0.2 (tomato) indicating that the presence of the endophytes mitigated the mortality risk of both species even in control conditions.

**Table 1:** Parameters of the regressions of the Cox proportional-hazards models.

Factor	Lettuce		Tomato	
	*β* ± SE	HR (*e*^*β*^)	*β* ± SE	HR (*e*^*β*^)
E+	−0.52 ± 0.45	0.7 ± 0.3	−1.04 ± 0.58	0.4 ± 0.2
salt stress	0.75 ± 0.34	2.2 ± 0.7	1.13 ± 0.36	3.3 ± 1.1
E+/salt stress	0.01 ± 0.55	1.2 ± 0.6	0.35 ± 0.67	1.7 ± 1.0

For both species the three influencing factors (i) E+ (with endophytes in 50 mM NaCl), (ii) salt stress (without endophytes in 150 mM NaCl), and (iii) E+/salt stress (with endophytes in 150 mM NaCl) were evaluated against the control condition (without endophytes in 50 mM NaCl). The models are based on 200 individuals for each species monitored for 12 weeks and 61 lethal events for lettuce and 56 for tomato. HR: hazard ratio, SE: standar error. The variance of HR was calculated on the basis of the 95% confidence interval of *β* ([*β*-SE..*β* + SE] → [HR_min_..HR_max_]).

### Ecophysiological Traits

**Figure 3:**
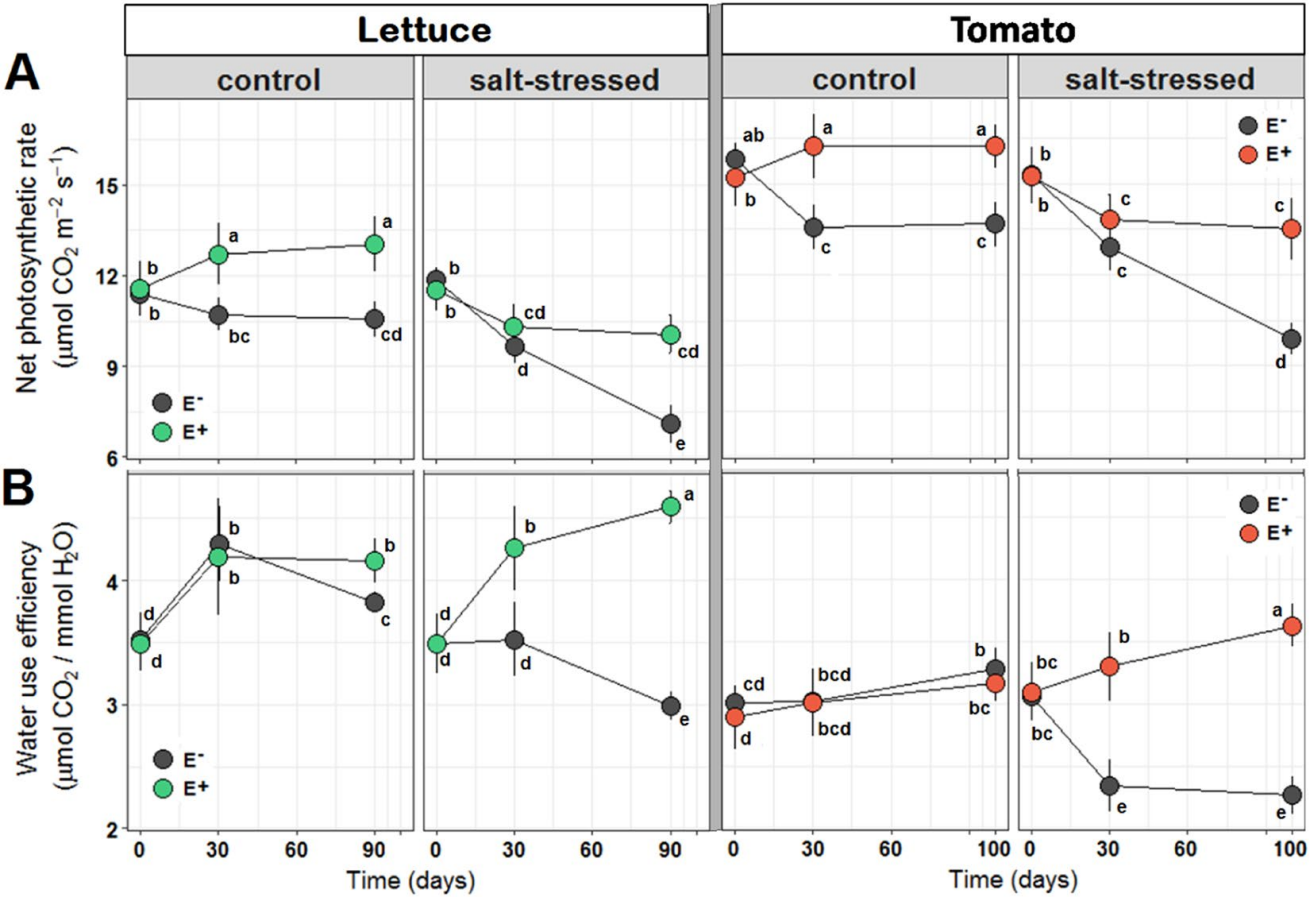
Effect of saline treatment (50 mM NaCl, control, vs. 150 mM NaCl, salt-stressed) and fungal inoculation (E- vs. E+) on the net photosynthetic rate (*A*_*max*_; A) and the water use efficiency (WUE; B) of lettuce (left) and tomato (right). Different lowercase letters indicate, for each species separately, significant differences between treatments (Tukey HSD tests; α = 0.05). Values are means ± SD (*n* = 20).

To get an idea on how the endophytes improved the fitness of the plants, we analyzed their photosynthetic capacity (net photosynthetic rate A_max_; Fig. 3A) and estimated the water-use efficiency (WUE; Fig. 3B) for photosynthesis as the ratio between photosynthetic rate and transpiration. WUE is an indicator of plant water stress in a microenvironment because an increase in WUE is usually induced by a decrease in water availability^46^.

In both crops without endophytes, salt stress reduced significantly A_max_ (−33% in lettuce and −28% in tomato at the end of the cultivation period) and WUE (−22% in lettuce and −31% in tomato) (Fig. 3B; Table S1). However, when the stress was applied to plants with endophytes, the negative effect of salt stress on the net photosynthetic rate was compensated (−4% in lettuce and −1% in tomato) and the WUE was even improved (+20% in lettuce and +11% in tomato). The positive effect of the endophytes was more pronounced in stress conditions. While their inoculation resulted in an increase of A_max_ of +24% (lettuce) and +19% (tomato) in control conditions, this value increased to +43% (lettuce) and +37% (tomato) in salt stress conditions. The same applies to the WUE, with only marginal variations in low salt, and a significant increase of +54% (lettuce)/+60% (tomato) in salt stress (Fig. 3B). The increase of the net photosynthetic rate in control conditions indicates that the endophytes alone induced already a partially latent stress-response in the crop plants. This initial response then largely compensated for the inhibitory effect of salt stress on the net photosynthetic rate and overcompensated for the detrimental effect on WUE (Fig. 3A,B). Thus, the presence of the endophytes improved plant fitness in salt stress conditions by mitigating the negative effects on an efficient energy production.

### Nutrient content

As next, we analyzed in which way salt stress affected the content of the key nutrients nitrogen, phosphorus and potassium in the crops (Table 2). In the absence of endophytes, the application of salt stress resulted in an increase of the potassium content and a decrease of the nitrogen and phosphate content in both crops. The presence of endophytes mitigated the inhibitory effect on the nitrogen content and stimulated further the accumulation of potassium (in lettuce only, not in tomato), but had no significant influence on the phosphorus content. Even under low salt, the presence of the endophytes stimulated potassium (in tomato only, not in lettuce) and nitrogen accumulation (Table 2). The increased nitrogen and potassium content after endophyte inoculation pointed to an increased uptake of these elements from the soil.

**Table 2:** Concentration of nutrients (% of dry weight) in foliar tissues of lettuce and tomato plants grown for 90 and 100 days, respectively, under 50 mM NaCl (Control) and 150 mM NaCl (Salt-stressed) in the presence (E+) or absence (E−) of root fungal endophytes.

Species	Nutrient (%)	Control		Salt-stressed	
		E+	E−	E+	E−
Lettuce	Nitrogen	3.5 a (±0.16)	3.1 b (±0.13)	2.8 c (±0.18)	2.2 d (±0.22)
	Phosphorus	0.7 a (±0.11)	0.6 a (±0.09)	0.4 b (±0.11)	0.3 b (±0.11)
	Potassium	7.1 b (±0.81)	6.9 b (±1.07)	10.1 a (±0.54)	8.2 b (±0.89)
Tomato	Nitrogen	6.1 a (±0.14)	5.4 b (±0.17)	3.6 c (±0.21)	2.9 d (±0.19)
	Phosphorus	0.8 a (±0.11)	0.7 a (±0.12)	0.3 b (±0.11)	0.3 b (±0.09)
	Potassium	9.5 b (±0.33)	7.8 c (±0.42)	12.7 a (±0.39)	11.5 a (±0.41)

Values are means ± SD (n = 20). Different lowercase letters indicate significant differences among treatments for each nutrient by species combination (Tukey HSD tests; α = 0.05).

### Content of foliar Na^+^ and expression of vacuolar NHX exchanger

**Figure 4:**
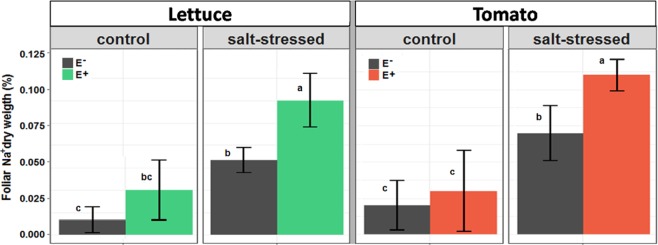
Foliar Na^+^ content of lettuce (left) and tomato plants (right) grown for 90 (lettuce) or 100 days (tomato) under 50 mM NaCl (control) and 150 mM NaCl (salt-stressed) in the presence (+E) or absence (−E) of root fungal endophytes. Values are means ± SE (n = 20). Different lowercase letters indicate, for each species separately, significant differences between treatments (Tukey HSD tests; α = 0.05).

The exposure of uninoculated plants to salt stress resulted in a significant increase of the foliar sodium content in both crops (Fig. 4). However, this increment was even larger in endophyte-inoculated plants. In other words, the endophyte-specific effect was more pronounced under high salt condition. Under low salt concentration -please, notice that the control condition contains already a significant amount of NaCl (i.e. 50 mM)-, the presence of endophytes provoked a slight increase in Na^+^ content in lettuce but not in tomato. In contrast, under salt stress conditions the endophytes caused a larger accumulation of sodium in leaves (Fig. 4).

**Figure 5:**
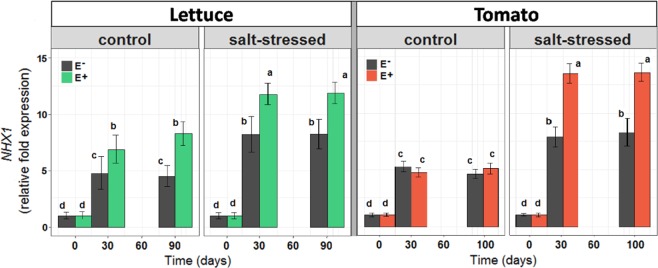
Effects of saline treatment (control vs. salt-stressed) and fungal inoculation (E− vs. E+ plants) on the expression level of the *NHX1* gene of lettuce and tomato plants along time. Different lowercase letters indicate, for each species separately, significant differences between treatments (Tukey HSD tests; α = 0.05). Values are means ± SE (n = 20).

Elevated cytosolic sodium concentrations are toxic for plants. In our results, however, the elevated Na^+^ content correlated with several improved physiological parameters. We therefore speculated that most of the sodium in the leaves has been sequestered in vacuoles, which is an efficient strategy to mitigate salt stress. One bottleneck for Na^+^-sequestration could be the availability of transport pathways into the vacuole. We therefore analyzed the expression level of the vacuolar Na^+^/H^+^ antiporter NHX1. For both species *L. sativa* and *S. lycopersicum* we identified the sequences of the genes homologous to *AtNHX1* and determined their expression levels by qRT-PCR in the four compared conditions (Fig. 5). It turned out that salt-stress alone already increased the *NHX1* expression levels. This upregulation was boosted further by the presence of the endophytes. Under low salt conditions the endophytes stimulated *NHX1* expression in lettuce but not in tomato. All these results correlated very well with the levels of Na^+^ accumulation in leaves. A higher *NHX1* expression was accompanied by a higher foliar Na^+^ content.

## Discussion

The two fungal endophytes originally isolated from Antarctic plants, *Penicillium chrysogenum* and *Penicillium brevicompactum*, had previously been found responsible for increasing yield under water shortage and salinity conditions in lettuce^7,11^. Besides improving plant physiological parameters (A_max_ and WUE), fungal endophytes also promoted the accumulation of the amino acid proline^7^, an osmotic molecule that has been previously suggested to mediate symbiont-conferred plant tolerance to water deficit^55,56^. More recently, it was found that the root fungal endophyte *Piriformospora indica* improved the ecophysiological performance of tomato plants under salinity by regulating Na^+^/K^+^ homeostasis and increasing the activity of antioxidant enzymes^10^. Antarctic fungal endophytes have been shown to be functional in the native Antarctic vascular plant *Colobanthus quitensis*^43^, but also when non-Antarctic plants were inoculated (e.g., *Flourensia thurifera*, *Puya berteroniana* and *Senna cumingii*^57,58^). Here we were able to find a relation between the improvements of individual plant performance and increased expression of the gene *NHX1* involved in ionic homeostasis. The accumulating evidence suggests that these endophytes are generalist for establishing functional symbiosis with even non-natural hosts. This finding might provide the foundation for using these organisms as biological tool to improve crops in hard situations.

We tested the potential of the Antarctic root-fungal endophytes *P. chrysogenum* and *P. brevicompactum* to improve the tolerance of two cultivated species (i.e. lettuce and tomato) to high level of salt-stress. Here we observed that endophyte-inoculated plants produced more total biomass than non-inoculated counterparts. Although sodium caused a significant reduction in plant size, this negative effect was more pronounced on non-inoculated plants. For both host species, the survival probability over the experimental time, was higher for inoculated plants than for non-inoculated plants. Like the effect on biomass, the beneficial effect of the root fungal endophytes on this latter response variable was hierarchically more evident under high sodium in the soil. The context-dependence of symbiosis-outcomes are similar across plant species and microorganism types^26^. However, in order to predict the outcome of a symbiotic interaction under a given environmental context, it turns out to be critical to know the changes caused by the microorganism on the plant morphology and physiology. For example, root endophytes can induce changes in plant root architecture by deploying new roots toward less salty soils^59,60^. Although we did not assess morphological changes in the root system of the plants, the greater relative growth at the end of the experiments could be at least partly attributed to the positive effect of the endophytes on root functionality (e.g., higher water up-take). Improved membrane functionality, higher concentration of osmotically active molecules and increased antioxidant capacity produced or modulated by fungal endophytes could explain the ability of plants to grow even under stress conditions^7,32^.

Besides being a source of bioactive secondary metabolites, symbiotic microbial symbionts can reprogram gene expression of the host to adjust the plant phenotype for improved tolerance to abiotic stress factors^32,61,62^. Particularly under salt stress, the beneficial effects of the Antarctic fungal endophytes became most evident on plant growth and survival. The inoculation of roots with fungal endophytes changed the expression of the *NHX1* gene in leaves of the two crop species. Although this gene may have different isoforms depending on the species^63^, it is involved in several functions related to the maintenance of the ionic balance and cell turgor^10^. High activity of NHX proteins (Na^+^/H^+^ antiporters integrated in vacuolar membranes) are associated with the capacity of accumulating Na^+^ inside vacuoles and, in turn, with a higher tolerance of the plant to toxic sodic salt soils^64^. We observed that in high-sodium soil, endophyte-inoculated plants were able to accumulate higher concentrations of Na^+^ in leaves compared to non-inoculated plants. The correlation of the up regulation of the vacuolar Na^+^/H^+^ antiporter NHX1 with increasing soil salt levels and with increasing accumulation of Na^+^ in leaves of endophyte-inoculated plants suggests a larger density of these transporters sequestering Na^+^ in vacuoles^65^. This endophyte mediated mechanism would allow plants to maintain ionic homeostasis and capacity to grow under high levels of sodium in the soil. As we only measured the expression associated to one of the NHX possible isoforms^63^, we can not specify whether this endophyte-mediated effect is general on all the isoforms or whether it is specific for NHX1. However, in the context of other publications reporting similar patterns and results (see e.g.^10,66^), our findings suggest that the effects of root fungal endophytes on plant tolerance to salt are general.

Central to any biotechnological breeding strategy on crop plants is that any change at the molecular level translates into improved physiological activity and ultimately, impacts on plant biomass accumulation. Here we observed that endophyte inoculated plants that showed an increased expression of the *NHX1* gene, displayed also higher photosynthetic capacity and water used efficiency. Compared to non-inoculated plants, the positive effects of the inoculated root endophytes on plant photosynthesis and WUE were more apparent under salinity conditions and as plants became bigger. A higher expression of NHX1 might be necessary but is not sufficient for a higher vacuolar Na^+^ transport rate, as such transport consumes energy^67,68^. Therefore, a positive feedback between different processes could explain the overall better performance of plants under stress. The improved photosynthetic rates and reduced transpiration rates of endophyte-inoculated plants, would be essential for maintaining successful sequestration of Na^+^. A reduced transpiration rate decreases the passive influx of Na^+^ ions into the roots^69^ and thus minimizes the energy required for Na^+^ extrusion and/or sequestration. An improved photosynthesis rate can contribute to cover the increased energy demand for driving the H^+^-ATPases and PPases that need to build up and maintain the electrical and proton gradients for an efficient Na^+^/H^+^ antiport for Na^+^ sequestration. Thus, lettuce and tomato plants were stimulated by the Antarctic root endophytes to orchestrate a diverse set of physiological parameters in a concerted action which finally allowed them to cope successfully with a salt stress condition. We can, therefore, conclude that the two endophytes *Penicillium brevicompactum* and *P. chrysogenum* mitigated salt stress in lettuce and tomato, provoking an efficient gain of carbon through a higher photosynthetic energy production and controlling the negative effect of sodium ions, likely by sequestration in vacuoles.

The growing demand for goods, overexploitation of agricultural lands and global climate change have promoted a rapid expansion of saline soils worldwide, threatening regular practices of agriculture and food security^44^. Under such scenarios, the physiological and biochemical functions of both wild and cultivated plant species are impaired causing severe yield losses^59^. Although it still needs to be tested at real production scales, harnessing fungal endophyte symbionts of plants appears to be a suitable strategy not only for improving crop yields but also for the design of environmentally friendly agricultural strategies^5,6,11,14^.

## Supplementary information

Supplementary information.
