# Supplementary material for: Antarctic root endophytes improve physiological performance and yield in crops under salt stress by enhanced energy production and Na+ sequestration
Source: Sci Rep. 2020 Apr 2;10:5819. doi: 10.1038/s41598-020-62544-4 (PMC7118072; doi:10.1038/s41598-020-62544-4)
Supplement: Supplementary file 1 — Supplementary information. [file 41598_2020_62544_MOESM1_ESM.docx]

**Supplementary material**

**Table S1.** Results of repeated measures ANOVA’s assessing the effects endophyte inoculation (E) and saline stress (S) on the net photosynthetic rate (*A_max_*), water use efficiency (WUE) and *NHX1* gene expression in lettuce and tomato plants. Significant probability values (*p* < 0.05) are highlighted in red. The results for the factor “time” and its interactions with E and S are not included since all of them resulted highly significant (*p* < 0.001) for both species in the three evaluated traits.

| **Species** | **Response Variable** | **Source of variation** | **df** | **SS** | **MS** | ***F*** | ***P*** |
| --- | --- | --- | --- | --- | --- | --- | --- |
| **Lettuce** | **Photosynthesis**  **(*A*_max_)** | Endophyte (E) | 1 | 79.07 | 79.07 | 133.04 | **< 0.0001** |
|  |  | Stress (S) | 1 | 112.81 | 112.81 | 189.82 | **< 0.0001** |
|  |  | E x S | 1 | 2.38 | 2.38 | 4.06 | 0.0512 |
|  |  | Residuals | 56 | 33.28 | 0.59 |  |  |
|  | **WUE** | Endophyte (E) | 1 | 10.78 | 10.78 | 215.43 | **< 0.0001** |
|  |  | Stress (S) | 1 | 2.22 | 2.22 | 44.54 | **< 0.0001** |
|  |  | E x S | 1 | 7.67 | 7.67 | 153.43 | **< 0.0001** |
|  |  | Residuals | 76 | 3.80 | 0.05 |  |  |
|  | **Gene expression**  **(*NHX1*)** | Endophyte (E) | 1 | 3.21 | 3.21 | 68.4 | **< 0.0001** |
|  |  | Stress (S) | 1 | 4.59 | 4.59 | 97.83 | **< 0.0001** |
|  |  | E x S | 1 | 0.40 | 0.40 | 8.59 | **0.0097** |
|  |  | Residuals | 16 | 0.75 | 0.04 |  |  |
| **Tomato** | **Photosynthesis**  **(*A*_max_)** | Endophyte (E) | 1 | 104.42 | 104.42 | 293.73 | **< 0.0001** |
|  |  | Stress (S) | 1 | 124.51 | 124.5 | 350.21 | **< 0.0001** |
|  |  | E x S | 1 | 0.02 | 0.02 | 0.04 | 0.8321 |
|  |  | Residuals | 56 | 19.91 | 0.36 |  |  |
|  | **WUE** | Endophyte (E) | 1 | 7.385 | 7.38 | 120.61 | **< 0.0001** |
|  |  | Stress (S) | 1 | 0.81 | 0.80 | 13.15 | **0.0005** |
|  |  | E x S | 1 | 11.39 | 11.39 | 186.13 | **< 0.0001** |
|  |  | Residuals | 76 | 4.65 | 0.06 |  |  |
|  | **Gene expression**  **(*NHX1*)** | Endophyte (E) | 1 | 4.42 | 4.42 | 121.93 | **< 0.0001** |
|  |  | Stress (S) | 1 | 12.59 | 12.59 | 347.24 | **< 0.0001** |
|  |  | E x S | 1 | 2.99 | 2.99 | 82.63 | **< 0.0001** |
|  |  | Residuals | 16 | 0.58 | 0.036 |  |  |
